# PSICA: Decision trees for probabilistic subgroup identification with categorical treatments

**DOI:** 10.1002/sim.8308

**Published:** 2019-06-27

**Authors:** Oleg Sysoev, Krzysztof Bartoszek, Eva‐Charlotte Ekström, Katarina Ekholm Selling

**Affiliations:** ^1^ Department of Computer and Information Science Linköping University Linköping Sweden; ^2^ Department of Women's and Children's Health Uppsala University, Akademiska Sjukhuset Uppsala Sweden

**Keywords:** bootstrap, decision trees, personalized medicine, random forest, subgroup discovery

## Abstract

Personalized medicine aims at identifying best treatments for a patient with given characteristics. It has been shown in the literature that these methods can lead to great improvements in medicine compared to traditional methods prescribing the same treatment to all patients. Subgroup identification is a branch of personalized medicine, which aims at finding subgroups of the patients with similar characteristics for which some of the investigated treatments have a better effect than the other treatments. A number of approaches based on decision trees have been proposed to identify such subgroups, but most of them focus on two‐arm trials (control/treatment) while a few methods consider quantitative treatments (defined by the dose). However, no subgroup identification method exists that can predict the best treatments in a scenario with a categorical set of treatments. We propose a novel method for subgroup identification in categorical treatment scenarios. This method outputs a decision tree showing the probabilities of a given treatment being the best for a given group of patients as well as labels showing the possible best treatments. The method is implemented in an R package **psica** available on CRAN. In addition to a simulation study, we present an analysis of a community‐based nutrition intervention trial that justifies the validity of our method.

## 1 INTRODUCTION

It is very common that randomized trials are performed to investigate the efficiency of a new treatment. In these trials, a new treatment is compared to a control treatment, and if the new treatment is shown to be more efficient than the control it is suggested to be used on a population‐wide level. Alternatively, in confirmatory subgroup analysis, effect of the treatment is investigated in the prespecified subgroups.^1^

Methods from personalized medicine^2^ have drawn a lot of attention in medical and statistical literature.^3^ These methods aim to identify and propose the best treatments to a patient with given characteristics (medical history). This clearly might lead to more efficient therapies than those proposed by confirmatory randomized trials. A lot of methods from personalized medicine are related to applications in genetics, ie, these methods detect treatments that persons with specific genetic biomarkers benefit of. From a statistical perspective, this typically reduces to a high‐dimensional regression problem with binary input variables indicating the absence or presence of corresponding genetic biomarkers.

Another important category of personalized medicine is subgroup identification, a comprehensive survey of methods from this category is available in the literature.^4^ The methods from this category identify subgroups of patients, which benefit from the same treatments, and this identification can be based on the characteristics of various natures (binary, categorical, real valued). Subgroup discovery methods can be applied to various experimental designs, including randomized clinical trials.^5^

Some personalized medicine methods are devoted to modeling optimal treatment regimes (OTR).^6^, ^7^, ^8^, ^9^, ^10^, ^11^, ^12^ The primary purpose of these methods is to determine the optimal treatment for a given patient rather than detecting subgroups having similar treatment effects. While some methods for the OTR prediction are *black‐box* models, many approaches were proposed to deliver interpretable optimal treatment decisions.^8^, ^9^, ^10^, ^11^, ^12^ Compared to the subgroup identification methods, the OTR methods search for a single optimal treatment for a given patient or groups of patients.

We focus on the subgroup identification methods that are inspired by decision tree structures. Decision trees are easily interpretable, which makes them very convenient for policy making. A decision‐maker is thus not only able to see what treatments are recommended but also which patient characteristics this recommendation is based on. Some comparative analysis of such methods is reported.^13^

It appears that the majority of subgroup identification methods focus on two‐arm trials, ie, when the treatment set is binary (control/treatment). Methods such as QUINT,^14^ Virtual Twins (VT),^5^ Interaction Trees (IT),^15^ and SIDES^16^ are able to identify subgroups in the binary scenario. Being very efficient in some settings, all of these methods have peculiarities that in some situations can be considered as limitations. Most importantly, all these methods except QUINT are focused on finding the groups when the treatment is better than the control, but they ignore the situations when the inverse is true (called qualitative interaction). Among other peculiarities/limitations, one may mention inability of processing continuous outcomes (eg, VT), nonprobabilistic nature of the algorithm (eg, QUINT), overlapping subgroups (SIDES), providing information about the mean difference in outcome within a subgroup rather than stating the probability that one treatment is better than the other one (IT). A few methods go behind the binary scenario: recently, a method treating continuous treatments (ordered by dose) was proposed.^17^

When trials are performed with a categorical set of treatments, no subgroup identification method exists that aims at finding subgroups and predicting which set of treatments is the best. In principle, model‐based (MOB) trees^18^ can be used to explain the dependence of the outcome on the medical history variables (characteristics) and the treatment variable. However, because this method tries to explain the outcome itself rather than the dominance of some treatments (prognostic variable problem^19^), very long trees might be needed to identify necessary subgroups. This makes conventional MOB trees very hard to use in practical policy making. It is also possible to apply the Gi method^19^ to a scenario with a categorical set of treatments, but this method outputs mean outcomes per treatment and subgroup rather than specifying the best treatments. It means that when two or more treatments have the same expected outcome, this method would not be able to identify such a situation due to randomness in the observed outcome mean.

We propose a novel method that is able to handle a scenario with categorical treatments (ie, when two or more different types of treatments are considered). We call this method Probabilistic Subgroup Identification for CAtegorical (PSICA) treatments. Our method is designed for randomized controlled trials and continuous outcome variables. We believe that it is of great importance for a subgroup identification method to provide statistical guarantees in the form of the probabilities of a treatment being the best for a given subgroup and, when data are not sufficient for a reliable decision, to state that there is no statistical guarantee that one of the treatments is more appropriate than the others. This differentiates our method from the OTR approaches and from many existing subgroup identification methods. Accordingly, our method first uses random forests to compute the probabilities that a treatment is the best for a given patient, and then these probabilities are summarized by a decision tree in which each terminal node shows probabilities for a treatment to be the best and the label showing most likely treatments. When all probabilities are large enough within a node, its label may contain all treatments, which is equivalent to saying “I don't know which treatment is the best” (ie, collect more data).

**Figure 1:**
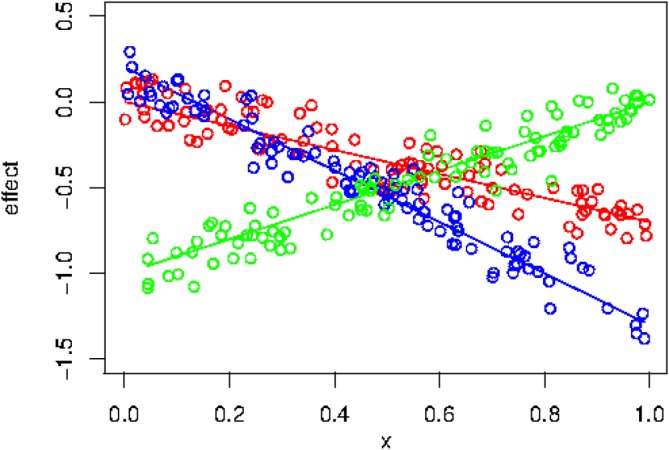
The outcomes for three treatments τ_1_=A, τ_2_=B, and τ_3_=C are generated as y(x,τ_1_)=−0.7x+ϵ (red), y(x,τ_2_)=−1.5x+0.2+ϵ (blue), y(x,τ_3_)=x−1+ϵ (green). Error term ϵ was generated as N(0,0.01) [Colour figure can be viewed at wileyonlinelibrary.com]

**Figure 2:**
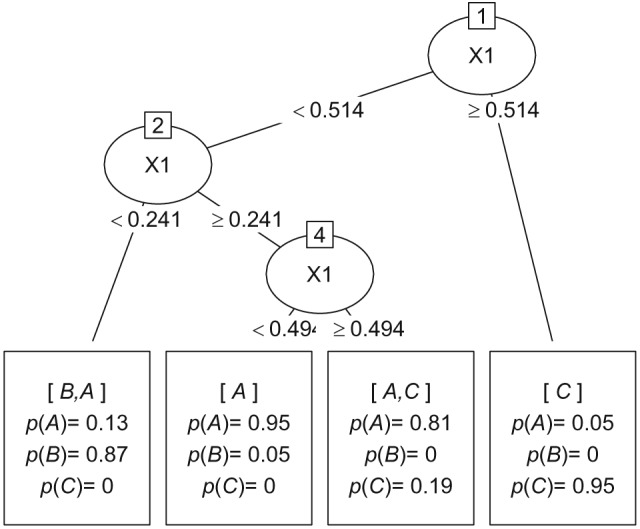
A PSICA tree showing subgroups, the probabilities of treatments being the best and labels containing the most likely optimal treatments

As an example, consider three treatments in which the outcomes are linear with respect to characteristics *x*. Figure 1 demonstrates such an example and some amount of observations corresponding to this setting. If the highest outcome implies the best treatment, treatment B is supposed to be the best for smaller values of *x*, treatment A should be the best for moderate *x*, and treatment C should be the best for the larger *x*. However, for smaller *x*, treatments A and B do not differ so much, which means that a subgroup discovery method would probably have a hard time to identify one best treatment. Figure 2 demonstrates the result of application of the PSICA method to these data. It clearly illustrates that the subgroups are identified in the way that was expected.

In our numerical experiments, we compare PSICA with QUINT when there are two treatments. We choose the QUINT method for comparisons because it is the only existing method not only capable of choosing the best treatment among two alternatives but also stating when the treatments are equivalent. In addition, we use PSICA to perform subgroup identification for the MINIMat trial^20^ that was conducted in Matlab subdistrict, rural Bangladesh and contained 6 categorical interventions (treatments).

In Section 2, we present the PSICA method. In Section 3, we present our numerical simulations and a real case study. Section 4 contains conclusions and discussion.

## 2 PSICA TREES

The problems of subgroup identification and some notation are introduced first. Given a data set D={(X_i_,Y_i_,t_i_),i∈1,…,n}, where X_i_=(X_i1_,…,X_ip_) is a set of characteristics (input variables, predictors) for patient i, Y_i_ is the outcome of the given treatment t_i_, where t_i_ is one of the treatments that belong to the set $T=\{\tau_{1},\text{…},\tau_{m}\}$. We assume that X_i_ values were obtained as a realization of a random variable x with components x ^1^,…,x ^p^. The response Y(x,τ), called a potential outcome (or simply outcome), is an outcome of a given treatment τ, and we assume that all treatments are possible to use for any patient. In practice, a patient with some characteristics X_i_ is assigned to only one of the treatments t_i_, and the outcome Y_i_ is observed. The remaining Y(x,τ), x=X_i_,τ≠t_i_ are normally not available. However, the observed outcome (X_i_,Y_i_,t_i_) is related to the potential outcomes as

$$Y_{i}=\overset{m}{\underset{j=1}{\sum }}Y(X_{i},\tau_{j})\cdot I(\tau_{j}=t_{i}),$$

where I(z) is equal to one when z is true and zero otherwise.

In randomized controlled trials, the probabilities of assigning a patient with characteristics x to different treatments do not depend on these characteristics. Assuming in addition that the treatment status of a patient does not affect potential outcomes of other patients and that there are no hidden versions of the treatments,^21^ the expected potential outcome becomes equal to the expected observed outcome per treatment, ie, E(Y_i_|x=X_i_,τ=t_i_)=E(Y(X_i_,t_i_)).

We assume that Y(x,τ)=f (x,τ)+ϵ, where f (x,τ) is the expected potential outcome for a given x and τ. In agreement with the previous assumptions, the error terms ϵ are assumed to be independent between the patients and also independent between different treatment options of the same patient. The input variables may be categorical, ordinal or real valued, and the outcome is considered to be real valued.

In a binary setting, ie, when $T=(\tau_{1},\tau_{2})$, the subgroup identification problem can be defined as finding subgroups G such that

$$\pi (G,\tau_{2},\tau_{1})=p\left(Y(x,\tau_{2})>Y(x,\tau_{1})\;|\;x\in G\right)>1-\alpha ,$$

where α is some risk level, eg, 0.05. This means that it is of interest to find subgroups of patients for which the second treatment is significantly better than the first one (which typically is a control treatment). Another interesting scenario is a qualitative subgroup identification, which means that the interesting subgroups are either those having π(G,τ_2_,τ_1_)>1−α or those satisfying π(G,τ_1_,τ_2_)>1−α.

When there are more than two treatments, the subgroup identification problem can be defined as follows: identify groups G and subsets of treatments T⊂T such that

$$p\left(Y(x,\tau^{\prime })>Y(x,\tau^{\prime \prime })\;|\;x\in G,\tau^{\prime }\in T,\tau^{\prime \prime }\in T\setminus T\right)>1-\alpha .$$

It means that we want to either identify which treatments are useful and can be prescribed to a patient (treatments from T) or which treatments are useless for this subgroup and should not be given to these patients (treatments from T\T). Note that we require T≠T because otherwise T∖T becomes empty and (X,τ ′ ′) becomes undefined.

The PSICA trees partition the input space into nonoverlapping regions and provide a label for each region and a probability distribution on the set {τ_1_,…,τ_m_} specifying how likely it is that a given treatment is the best one for the group of patients characterized by the input values from this region. The PSICA tree computation consist of two steps: estimation of distributions and growing the PSICA tree.

The first step of PSICA tree computation implies estimating distributions π_k_(x), which is a probability that the treatment τ_k_ is better than all alternative treatments for a given x. To estimate π_k_(x),k=1,…,m by simulation, we need to be able to generate samples from the joint distribution p(Y(x,τ_1_),…,Y(x,τ_m_)). This distribution shows how likely it is that if a patient with characteristics x receives treatment τ_1_, then the outcome will be Y(x,τ_1_), and if the same patient receives τ_2_, then the outcome will be Y(x,τ_2_), etc. If we are able to generate some number of samples $Y^{b}=(Y_{1}^{b}(x),\text{…},Y_{m}^{b}(x)),b=1,\text{…},B$ from this distribution, then π_k_(x) can be estimated as

(1) $$\pi_{k}(x)=\frac{1}{B}\overset{B}{\underset{b=1}{\sum }}I\left(Y_{k}^{b}(x)>\underset{j=1,\text{…},m,j\neq k}{\max}Y_{j}^{b}(x)\right).$$

To generate samples from p(Y(x,τ_1_),…,Y(x,τ_m_)), we divide data D into subsets D_k_={(X_i_,Y_i_,t_i_):t_i_=τ_k_,(X_i_,Y_i_,t_i_)∈D} for all k=1,…,m. Each subset D_k_ corresponds to one of the treatments. The partitioned data are further used to generate samples Y ^b^ by method 1 or method 2.

Method 1 implies that B data sets $D_{k}^{b},b=1,\text{…},B$ are constructed by bootstrapping observations from D_k_, we denote it as $D_{k}^{b}∼\mathrm{Bootstrap}(D_{k})$. Afterwards, a machine learning model $M_{k}^{b}(x)$ is fit to each $D_{k}^{b}$ by using y as response variable and x as the set of predictor variables. We propose to use conditional inference random forest^22^ models but in principle any other machine learning (regression) model can be employed. Finally, samples $Y^{b}=(Y_{1}^{b}(x),\text{…},Y_{m}^{b}(x))$ for any given x are generated as $Y_{k}^{b}(x)=M_{k}^{b}(x),k=1,\text{…}m,b=1,\text{…},B$.

Method 2 implies fitting a machine learning model to each D_k_, estimating the prediction M_k_(x) and then estimating the variance $\sigma_{k}^{2}(x)$ of prediction by using the bias‐corrected infinitesimal jackknife,^23^ see formula (7) in the corresponding paper. Finally, components $Y_{k}^{b}(x)$ of the samples are generated from a normal distribution with mean M_k_(x) and variance $\sigma_{k}^{2}(x)$ for each b=1,…,B. Estimation of π_k_(x) is summarized in Algorithm 1.

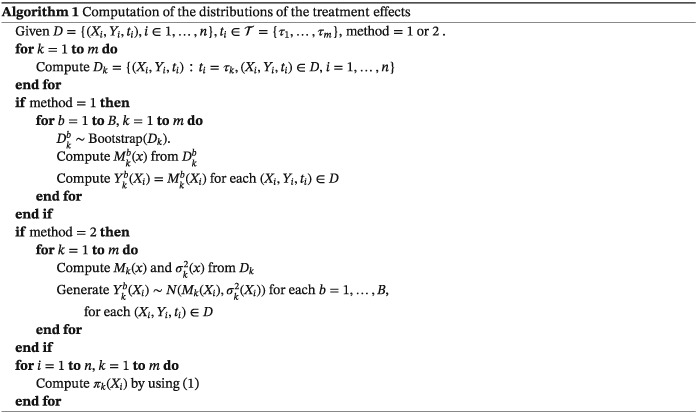

In order to make a choice between method 1 and method 2 in a practice, the following arguments can be considered. Method 1 is based on the bootstrap principle, which aims to approximate the sampling process from the true data generating distribution by sampling from the data set instead. This might lead to biased estimates and high costs in terms of computational time. However, when the true outcome distribution is not Gaussian, method 1 may still be much less biased than method 2, which assumes the normality of the outcome distribution.

The second step of PSICA tree computations implies growing a tree summarizing the probabilities π_k_(X_i_) in such a way that interesting subgroups are discovered. We suggest two alternative methods for the tree growing process: method A and method B. Method A requires growing a large tree and then letting a user to prune it until interpretable applied results are achieved and at the same time, the tree becomes small enough to be used for policy making. Random forests are known to be very flexible models that are robust to overfitting,^24^ which means that increasing the number of trees in the forests usually decreases the bias in estimation of the outcomes without increasing the variance. At the same time, increasing the number of bootstrap samples B decreases the variance in π_k_(x). Accordingly, for sufficiently large number of trees in the forests and for a sufficiently high number of bootstrap samples B, the probabilities π_k_(x) are expected to have small bias and small variance. However, it can be hard to judge whether the settings used by a user are sufficiently large, which means that there might be a risk for producing spurious subgroups. To remedy this problem, we suggest method B, which implies early pruning of the tree (prepruning) that guarantees that fewer spurious results are detected. However, since this method is based on hypothesis testing, there is a risk that some interesting subgroups are not found.

Method A employs standard decision tree growing principles.^25^ More specifically, a data set Δ_0_={(X_i_,P_i_),P_i_=(π_1_(X_i_),…,π_m_(X_i_))} with inputs X_i_ and a vector response P_i_ is constructed first. This data set is partitioned recursively by using various binary splitting rules R_j_ (constructed differently for real‐valued and categorical split variables) until some stopping criterion is met. This criterion might include constraints on the minimal amount of the observations in the node, maximal tree depth, and other criteria. To decide which splitting rules need to be used, the data set Δ that corresponds to a tree node before split R_j_ and the data sets after this split Δ_1_ and Δ_2_ are considered, and loss function values L_1_=L(Δ), L_2_=L(Δ_1_), and L_3_=L(Δ_2_) are computed. A splitting rule that maximizes information gain

(2) $$g(\Delta ,R_{j})=L_{1}-(L_{2}+L_{3})$$

is chosen to split the current node.

When no further split can be done, labels are assigned to the terminal nodes. In our settings, the following summary might be presented for a tree leaf corresponding to a data set Δ:

- Aggregated probabilities of each treatment being the best
  (3) $$\pi_{k}(\Delta )=\frac{1}{\left|\Delta \right|}\underset{(X_{i},P_{i})\in \Delta }{\sum }\pi_{k}(X_{i}),$$
  where |Δ| denotes the number of observations in Δ.
- A set of useless treatments $T_{u}$. The probabilities π_k_(Δ) are sorted in increasing order as $\left(\pi_{k_{1}}(\Delta ),\text{…},\pi_{k_{m}}(\Delta )\right)$ and m ′ is found such that $\sum_{i=1}^{m^{\prime }}\pi_{k_{i}}(\Delta )\leq \alpha$ and $\sum_{i=1}^{m^{\prime }+1}\pi_{k_{i}}(\Delta )>\alpha$, where α is a risk level (eg, α=0.05). The set $T_{u}$ is computed as $T_{u}=\{\tau_{k_{1}},\text{…},\tau_{k_{m^{\prime }}}\}$.
- A set of potential treatments
  (4) $$T_{p}=T\setminus T_{u}.$$

To enable successful subgroup identification, an appropriate loss function needs to be selected. To identify an appropriate function, it is important to consider how the resulting tree is going to be used in decision‐making. We assume that after a decision‐maker assigns the patient into one of the terminal nodes of the decision tree, the aggregated probabilities π_k_(Δ),k=1,…,m are compared, and the treatments from $T_{u}$ will be excluded by the decision‐maker. The remaining treatments are the potential treatments, and ideally, a further investigation of which one of them should be prescribed to a given patient will be performed. However, it is also very likely that the aggregated probabilities corresponding to treatments from $T_{p}$ will be used by the decision‐maker directly as an indicator of which treatment should be used.

Therefore, we define the loss function L(Δ) as the cost of assignment of the individuals represented by Δ to the treatments that they do not benefit from. More specifically, we define truncated probabilities as

(5) $$\begin{array}{ccc} {\hat{\pi }}_{k}(\Delta ) & =\frac{\pi_{k}(\Delta )}{\sum_{i\in T_{p}}\pi_{i}(\Delta )},k\in T_{p} & \\ {\hat{\pi }}_{k}(\Delta ) & =0,k\notin T_{p}, \end{array}$$

and therefore the cost of classifying a patient to a wrong treatment is

(6) $$\begin{array}{c} \overset{m}{\underset{k=1}{\sum }}\underset{j\in T_{p},j\neq k}{\sum }c_{kj}p(\text{Assigned to}\;\tau_{j}\;\text{given}\;\tau_{k}\;\text{is best})\cdot p(\tau_{k}\;\text{is best}), \end{array}$$

where {c_kj_,k,j=1,…,m} are costs of giving the patient treatment τ_j_ given that his/her best treatment is τ_k_. A simple set of cost values is a zero‐one cost: c_kj_=1 when k≠j and zero otherwise.

Equation (6) can be rewritten as

$$\overset{m}{\underset{k=1}{\sum }}\underset{j\in T_{p},j\neq k}{\sum }c_{kj}{\hat{\pi }}_{j}(\Delta )\cdot \pi_{k}(x)$$

and, by summing up the loss values for the observations within Δ, we obtain the following loss function:

(7) $$L(\Delta )=\underset{(X_{i},P_{i})\in \Delta }{\sum }\overset{m}{\underset{k=1}{\sum }}\underset{j\in T_{p},j\neq k}{\sum }c_{kj}{\hat{\pi }}_{j}(\Delta )\cdot \pi_{k}(X_{i}).$$

If the zero‐one loss is used, it is easy to show that (7) can be simplified as

(8) $$L(\Delta )=|\Delta |\overset{m}{\underset{k=1}{\sum }}\pi_{k}(\Delta )\cdot (1-{\hat{\pi }}_{k}(\Delta )).$$

Method B involves early stopping to avoid discovery of spurious subgroups. The tree growing procedure is identical to the first approach described above with the only exception that the information gain function g is modified in order to avoid splits that may generate spurious subgroups. More specifically, the modified information gain g ′ is defined as g ′(Δ,Δ_1_,Δ_2_)=g(Δ,Δ_1_,Δ_2_)·G(Δ_1_,Δ_2_), where G(Δ_1_,Δ_2_) is equal to one if the distributions Π_1_={π_k_(Δ_1_),k=1,…,m} and Π_2_={π_k_(Δ_2_),k=1,…,m} differ significantly and zero otherwise.

To compute function G, we perform a chi‐square test where we compare Π_1_ and Π_2_. For each Π_j_, we compute counts

(9) $$\left\{n_{kj}=\left\lceil \pi_{k}(\Delta_{j})\cdot |\Delta_{j}|\cdot \omega_{j}\right\rceil ,k=1,\text{…},m\right\},$$

where ω_j_ is an inflation factor defined as the standard deviation of the uniform distribution U[0,1] (which is equal to $1/\sqrt{12}$) divided by the standard deviation of {π_k_(X_i_):(X_i_,P_i_)∈Δ_j_}. The purpose of the inflation factor is to give higher weights to the distributions of π_k_(X_i_) that have low variance (and, thus, more confident). After the counts for Π_1_ and Π_2_ are computed, these counts are combined into a two‐way table, and the standard chi‐square test is performed. If its p‐value p_χ_ is lower than a risk level α, we set G=1 otherwise G=0.

The summary of the PSICA tree growing strategy is given in Algorithm 2.

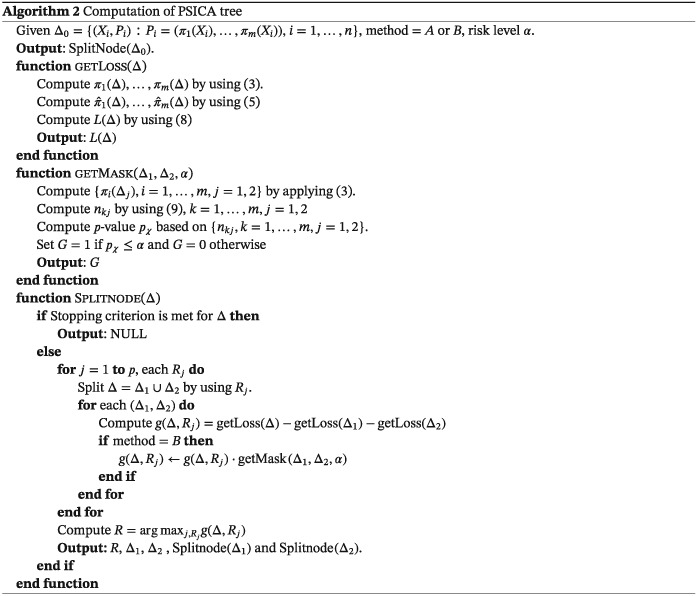

## 3 NUMERICAL EXPERIMENTS

Our PSICA method was implemented in an R package **psica**, which is available on CRAN.^26^ To evaluate the efficiency of the method, we tested it with the following models:

(10) $$\text{M1}:y(x,\tau )=(2\text{th}(2x)+3)I(\tau =\tau_{1})++(2\text{th}(x)+2.3)I(\tau =\tau_{2})+\epsilon ,$$

where ϵ∼N(0,0.8^2^) and th(x) is the hyperbolic tangent function. The variance in this and the following models was adjusted in such a way that the highest signal‐to‐noise ratio is approximately 10. Some properties of this function considered on the interval [−1,1] are that the function is relatively complex (ie, includes nonlinearities) and that τ_1_ is best in the entire interval while the effect of τ_1_ and τ_2_ becomes very similar around x=−0.5. Therefore, one can expect that subgroup identification methods should be able to either identify τ_1_ as the best treatment or they should be uncertain, for example, around x=−0.5 and especially for smaller data sets. The QUINT method is aimed at finding qualitative interactions, ie, it assumes that there exist regions where τ_1_ is better than τ_2_ and other regions where τ_2_ is better than τ_1_. It means that this method is expected to fail in finding such interactions when the data are generated from M1.

(11) $$\text{M2/M3}:y(x,\tau )=0.5I(x_{1}\geq 0\;\text{and}\;x_{2}\geq 0)I(\tau =\tau_{1})+0.5I(x_{1}<0\;\text{and}\;x_{2}<0)I(\tau =\tau_{2})+\epsilon ,$$

where ϵ∼N(0,0.2^2^) (M2) and ϵ∼Laplace(0,0.2^2^) (M3). These models contain qualitative interactions that are expected to be discovered by QUINT and also can be used to compare the effect of the error distribution (normal vs Laplace) on the performance of subgroup identification methods.

(12) $$\text{M4}:y(x,\tau )=\overset{40}{\underset{i=1}{\sum }}x_{i}+5x_{1}I(x_{1}>0.5)I(\tau =\tau_{1})+5I(x_{1}<0.5\;\text{and}\;x_{2}>0.5)I(\tau =\tau_{2})+\epsilon ,$$

where ϵ∼N(0,2^2^). This model is interesting to consider because it involves many variables in creating the main effect and a few variables that interact with the treatments.

(13) $$\text{M5}:y(x,\tau )=(-0.7x-0.7)I(\tau =\tau_{1})+(-1.5x-1.1)I(\tau =\tau_{2})+(x-1)I(\tau =\tau_{3})+\epsilon ,$$

where ϵ∼N(0,0.2^2^). Model M5 is similar to the model explained in Figure 1. It contains three treatments and it can thus not be processed by binary subgroup identification methods like QUINT. However, this model is good enough to study the behavior of PSICA model in a simple setting.

(14) $$\begin{array}{cc} \text{M6}:y(x,\tau ) & =\overset{40}{\underset{i=1}{\sum }}x_{i}+5x_{1}I(x_{1}>0.5)I(\tau =\tau_{1}\;\text{or}\;\tau =\tau_{2})+10(x_{1}<0\;\text{and}\;x_{0}{\;=}^{\prime }\;K1^{\prime })I(\tau =\tau_{3})+\epsilon , \end{array}$$

where ϵ∼N(0,2^2^), and $T=\{\tau_{1},\text{…},\tau_{4}\}$. In this model, there is a main effect and also complex treatment effects: one subgroup benefits from treatments τ_1_ and τ_2_ while another subgroup benefits from treatment τ_3_. None of the patients benefits from τ_4_. This model also includes a categorical variable x_0_ with four unique values, and this variable is important in defining one of the subgroups. This model can thus be regarded as good test of PSICA trees in real complex scenarios.

We perform the following numerical experiments 200 times. First, we generate data from models M1 to M6 with n observations, where n=300,900, or 1800 and a randomized treatment assignment, where each x component is generated as U[−1,1]. To make the correct subgroup identification even more difficult for the estimation algorithms, we add a number of irrelevant input variables generated as U[−1,1] to each data set: two variables for models M1, M2, M3, and M5, 160 variables for M4 and M6. In the next step, we perform subgroup identification by using PSICA (for M1 to M6) and QUINT (for M1 to M4). When computing PSICA trees, we use three alternatives: method m_1_ denotes PSICA trees with probabilities computed by the bias‐corrected infinitesimal jackknife (method 2 in Algorithm 1) and the number of variables per split in the random forest equals the total amount of input variables p, method m_2_ denotes PSICA trees with probabilities computed by the bias‐corrected infinitesimal jackknife and the number of variables per split in the random forest equal to $\sqrt{p}$, method m_3_ PSICA trees computed by the bootstrap approach (method 1 in Algorithm 1) and the number of variables per split in the random forest equal to $\sqrt{p}$. Method m_3_ is computed only for n=300 due to high computational burden. Other settings were specified as B=500, all PSICA trees used method B (see Algorithm 2) with α=0.05, number of trees in a forest equal to 100, minimal amount of observations for splitting the node in a tree equal to n/10 in the trees belonging to forests and n/5 in the PSICA trees. When computing QUINT trees (denoted as method m_4_), we use the bootstrap pruning and default settings specified in the corresponding R package.^27^

The performance of the methods is evaluated by computing the following metrics: accuracy (a), uncertainty (u), and suspect (s). Given that for each feasible x, a tree delivers the predicted best treatments $T_{p}$ while the true best treatments are T_p_, metrics a and u are defined as follows:

(15) $$\;a(D)=\frac{1}{n}\underset{(X,Y,T)\in D}{\sum }I(T_{p}(X)\subseteq T_{p}(X))$$

(16) $$u(D)=\frac{1}{n}\underset{(X,Y,T)\in D}{\sum }I\left(\left|T_{p}(X)|>|T_{p}(X)\right|\right),$$

where |S| denotes the number of elements in a set S. Accuracy values represent proportions of the correct predictions while the uncertainty values specify how uncertain the tree is. Note that a tree can in principle achieve 100% accuracy by predicting all treatments as a full set T, but it will also imply 100% uncertainty.

The suspect s(Δ) is defined as a the sum of the amounts of observations corresponding to the nodes that are immediately above the irrelevant splits divided by the sum of the amounts of observations corresponding to all nodes in the tree. Therefore, if an irrelevant variable is located in the top levels of the tree, the suspect value is expected to be high.

For PSICA trees, we also compute a measure, which we call decision accuracy. As it was discussed in Section 2, we assume that when a terminal node in the PSICA tree returns a set of potential treatments $T_{p}$, and this set contains more than one treatment, a decision‐maker is ideally supposed to make further investigations regarding which of these treatments should be given to a patient. However, it is also likely that the decision‐maker will use the aggregated probabilities shown in the corresponding tree node to make a decision. However, this might not be a good strategy in some situations. Suppose $T=\{\tau_{1},\tau_{2}\}$ and in the given tree node π_1_=0.45 and π_2_=0.55. Although treatment τ_2_ has a somewhat higher probability, it is clear that the model is quite unsure about which treatment is the best one for the group of patients associated with the given tree node. This means that, in this case, a further investigation is probably the most reasonable option. Assume though that the PSICA tree returns a set of truncated probabilities $\left\{{\hat{\pi }}_{k}(x),k=1,\text{…},m\right\}$ for a given x and the decision‐maker makes a decision as $\tilde{\tau }(x)∼\text{Multinomial}\;({\hat{\pi }}_{1}(x),\text{…},{\hat{\pi }}_{m}(x))$. Decision accuracy measures the proportion of the correct decisions in this scenario as

(17) $$\delta (D)=\frac{1}{n}\underset{(X,Y,T)\in D}{\sum }I\left(\tilde{\tau }(X)\in T_{p}(X)\right).$$

**Figure 3:**
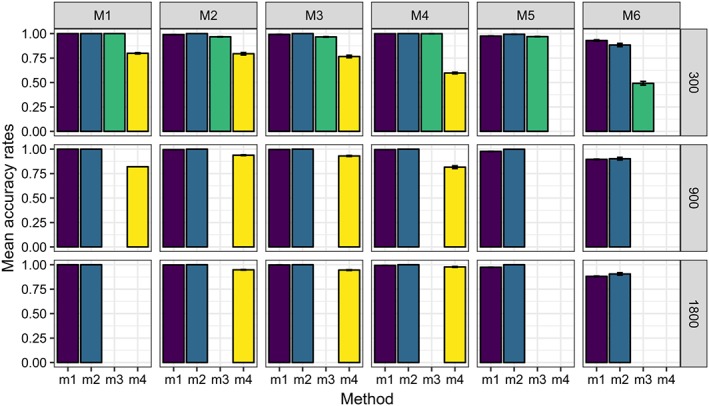
Mean accuracy rates (over 200 experiments) for different data models (M1 to M6) processed by four methods (m_1_ to m_4_). Standard error of the mean is specified by the whiskers. Each panel of the graph corresponds to some data model (defined by the column title) and some data size (defined by the row title) [Colour figure can be viewed at wileyonlinelibrary.com]

**Figure 4:**
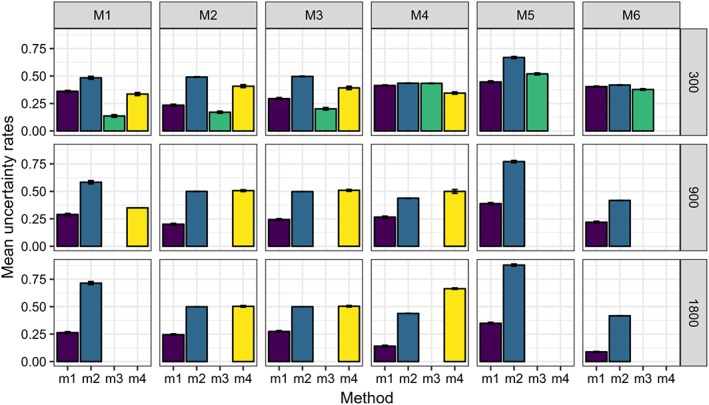
Mean uncertainty rates (over 200 experiments) for different data models (M1 to M6) processed by four methods (m_1_ to m_4_). Standard error of the mean is represented by the whiskers. Each panel of the graph corresponds to some data model (defined by the column title) and some data size (defined by the row title) [Colour figure can be viewed at wileyonlinelibrary.com]

**Figure 5:**
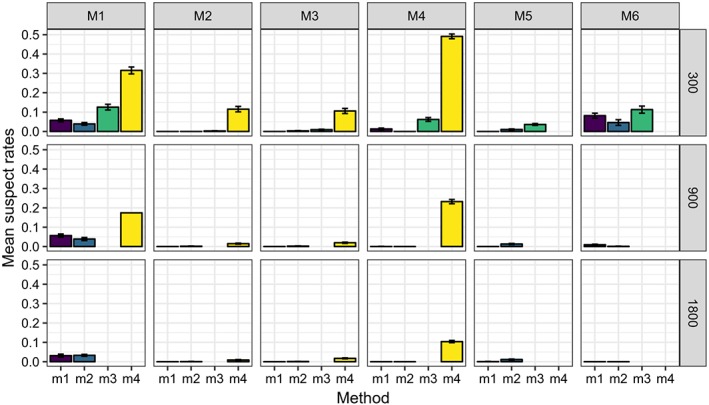
Mean suspect rates (over 200 experiments) for different data models (M1 to M6) processed by four methods (m_1_ to m_4_). Standard error of the mean is represented by the whiskers. Each panel of the graph corresponds to some data model (defined by the column title) and some data size (defined by the row title) [Colour figure can be viewed at wileyonlinelibrary.com]

**Figure 6:**
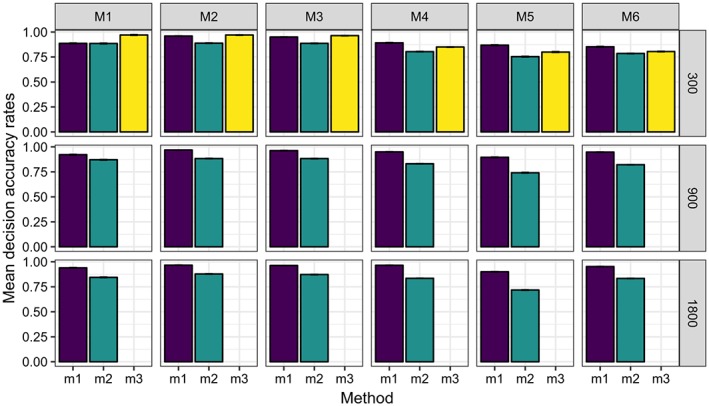
Mean decision rates (over 200 experiments) for different data models (M1 to M6) processed by four methods (m_1_ to m_4_). Standard error of the mean is represented by the whiskers. Each panel of the graph corresponds to some data model (defined by the column title) and some data size (defined by the row title) [Colour figure can be viewed at wileyonlinelibrary.com]

Figures 3, 4, 5, and 6 illustrate the results of our simulation experiments, and the underlying data tables are provided in the Appendix, see Tables A1, A2, A3, and A4.

It can be concluded that *m*_1_, *m*_2_, and *m*_3_ provide a similar accuracy, which is close to 100*%* in the majority of scenarios, both when binary treatments are used and when categorical scenarios are considered. Method *m*_4_ (QUINT) has lower accuracy values, especially for smaller data and when there are many irrelevant variables (model M4). Accuracies of models *m*_1_,*m*_2_,*m*_3_ also decrease when the data model is complex and there are many irrelevant predictors (model M6). By comparing the accuracies of methods *m*_1_‐*m*_3_ across M2 and M3, no noticeable difference can be detected, which indicates that PSICA trees are not so sensitive to the error distribution.

When comparing uncertainties, an interesting fact can be observed: allowing the conditional inference random forest to use all input variables at any split (method *m*_1_) leads to lower uncertainty rates than the setting $\sqrt{p}$ variables at any split (method *m*_2_), which is recommended in the literature for the random forests. This happens because the trees in the forests are grown by means of early stopping involving hypothesis testing: if there are no relevant variables in the randomly selected subset of input variables, the corresponding tree node will not be split further. It may lead to deficient trees in some cases. It can also be observed that uncertainty rates decrease with increasing sample size for model *m*_1_, while for models *m*_2_ and *m*_4_, these rates usually do not change much or sometimes increase. Noticeably, uncertainty rates of *m*_1_ are generally lower than those of *m*_2_ and *m*_4_, and the uncertainty rates of *m*_2_ are generally comparable to the rates of *m*_4_ with the two exceptions. The first exception is a simple model (M1) where *m*_4_ has high uncertainty rates. The second exception is a complex model (M4) where the *m*_2_ rates are lower than the *m*_4_ rates for larger *n*. The uncertainty rates of *m*_3_ are often lower than those of *m*_1_ indicating that applying bootstrap instead of the variance approximation might lead to better decisions. The price of this is a much higher computational time. Figure 5 illustrates that PSICA is good in finding relevant predictors: the suspect rates change approximately between 0 and 0.1. QUINT gives relatively low suspect rates for models M2 and M3. However, when a complex data model with many irrelevant predictors is processed (model M4), *m*_4_ appears to have problems in finding the relevant predictors. When the data model implies that one treatment is the best one for all observations (model M1), QUINT produces sometimes trees with a root node only (which are excluded from computations of the suspect) and sometimes produces trees with irrelevant splits. When *n*=1800, all computed QUINT trees contained only the root node, and therefore no suspect value is reported.

Figure 6 demonstrates that the decision accuracies for method *m*_1_ are often very high (0.8‐1.0), and they grow with increasing sample size. Method *m*_2_ has somewhat lower decision accuracies, which confirms our previous finding: using all input variables at any split leads to a better performance of PSICA trees. Decision accuracies generated by method *m*_3_ are comparable to the results obtained by *m*_1_.

**Figure 7:**
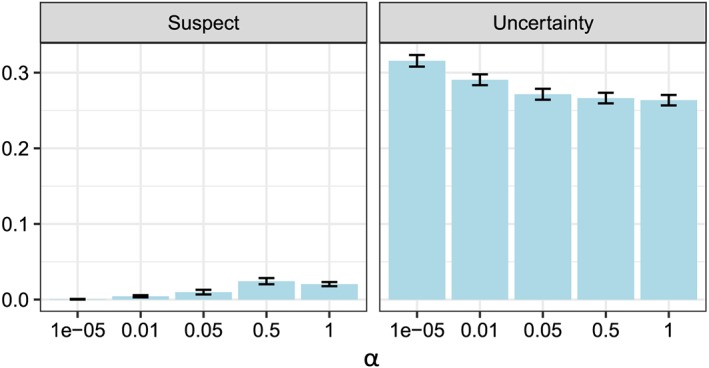
Mean uncertainty rates and mean suspect rates (over 400 experiments) for the data sets processed by m_1_). Standard error of the mean is represented by the whiskers [Colour figure can be viewed at wileyonlinelibrary.com]

To study the effect of the risk level *α* in the tree pruning algorithm (method B), we perform additional 400 simulation experiments. In each experiment, we randomly choose *n* from the set {300,900,1800} and the data model from {*M*1,…,*M*6}. A data set of size *n* is then generated according to the selected data model, and the generated data set is processed by the method *m*_1_, and accuracy, uncertainty, suspect, and decision accuracy values are computed. Method *m*_1_ was chosen because it had an overall high accuracy, relatively low uncertainty and low suspect rates in the previous experiments. The results are illustrated by Figure 7, and more detailed information is provided in Table A5.

The accuracy and decision accuracy rates were roughly the same for different *α* values, but the uncertainty and the suspect rates were more affected by the choice of *α*. Figure 7 illustrates that the suspect rates tend to increase when *α* increases. At the same time, uncertainty rates tend to decrease when *α* increases, which means that too low values of *α*, eg, *α*=10^−5^, are not optimal either. The value *α*=0.05 has relatively low uncertainty rates and relatively low suspect rates and thus can be recommended in practice.

In addition to the simulation experiments, we analyze the so called MINIMat data^20^ with the PSICA method. The MINIMat trial was conducted in the Matlab subdistrict, rural Bangladesh. In this area, 4436 pregnant women were enrolled between November 2001 and October 2003 to take part in the trial. The design and interventions of the MINIMat trial have previously been described in detail.^28^ Very briefly, pregnant women were individually and randomly allocated in a 2 by 3 factorial design into two prenatal food and three micronutrient supplementation groups. Food supplementation was promoted to start either in early pregnancy (E for early) or at the women's own liking (U for usual). The three micronutrient groups were: 30 mg of iron supplementation (X), 60 mg iron (Y), 30 mg of iron, 400 mg of folic acid, and 13 other micronutrients (Z). At enrollment and during pregnancy, characteristics of the women and their households were collected. In this example, 124 variables, such as maternal anthropometry, parity, education, morbidity, exposure to domestic violence, as well as household food insecurity and assets, during the time of pregnancy were included as inputs.

The outcome variable is the children's height‐for‐age z‐score at 54 months (HAZ), and the aim of PSICA tree analysis is to find out which interventions increase HAZ of the children. We computed a PSICA tree with pruning, *B*=1000, *α*=0.05, number of trees in a forest equal to 100, minimal amount of observations for splitting the node in a tree equal to 40 in the trees belonging to forests and equal to 60 in the PSICA tree.

**Figure 8:**
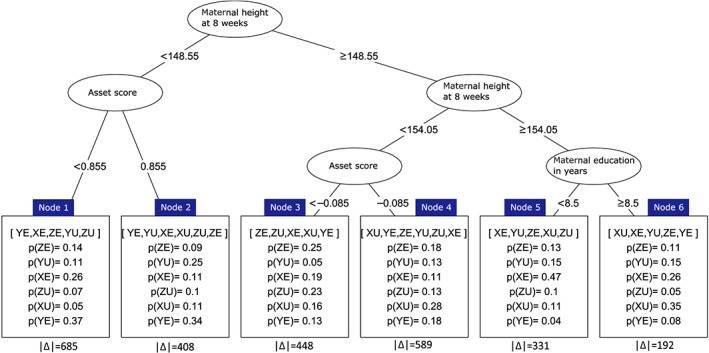
A PSICA tree showing subgroups and probabilities of various treatments for the MINIMat trial. Amounts of observations in the terminal nodes are represented by |Δ| [Colour figure can be viewed at wileyonlinelibrary.com]

Figure 8 shows that in four out of six nodes (Nodes 1, 2, 3, 5), supplementation options including early food supplementation had a larger probability of increasing HAZ at 54 m than the usual food supplementation, and this is in agreement with previous results of the trial,^29^ Table A3. Similarly, our finding that in three out of six nodes (Nodes 4, 5, and 6), a supplementation including 30 mg of iron, and in two out of six nodes (Nodes 1 and 2) supplementation containing 60 mg of iron had higher HAZ compared to multiple micronutrient supplementation is also in agreement with previous results,^29^ Table A3.

A result that has not been shown previously is that the optimal micronutrient supplementation varied with maternal height. Among the shortest women (Nodes 1 and 2), supplements containing 60 mg had the highest probability of a better HAZ in their offsprings. Among taller women (Nodes 3 to 6), supplements with a lower amount of iron (30 mg and MMS) had higher probabilities, and in three out of these four nodes, the optimal supplementation was 30 mg. While these differences in effects on young child height development have not previously been shown, they are biologically plausible in that shorter women are likely to have experienced more of nutrients deficiencies and thus larger nutrient requirements such as a larger dose of iron may be needed for optimal growth of their children. Maternal height has been shown to modify effect of micronutrient supplementation on other early life outcomes^30^ and it is reasonable to believe that it will also modify other later outcomes. Similarly, indicators of socioeconomic situation such as maternal education have been shown to modify effect of micronutrient supplementation on early life outcome^30^ and thus may also be of importance for young child height. The importance of iron for fetal, infant, and child growth has been shown in studies in low‐income settings^31^, ^32^ and iron supplementation has been highlighted as a key intervention to improve maternal and children's health.^33^

## 4 CONCLUSIONS AND DISCUSSION

In this work, we introduce PSICA trees. This is a novel method for subgroup identification in scenarios with categorical sets of treatments. Our numerical results illustrate that, with appropriate settings, PSICA trees provide high accuracies of prediction of the best treatments, and the method's uncertainty decreases with an increasing amount of data. At the same time, PSICA trees are easily interpretable and can therefore be used for policy making. The PSICA trees seem to be able to identify meaningful subgroups even when there are moderate mean effects from a lot of inputs, while in these cases, the QUINT method often fails to identify meaningful subgroups or it gives low accuracies. The PSICA trees are also able to handle cases when none of the treatments leads to a significantly better outcome than the other treatment: in this case, a noninformative tree (ie, in which all the treatments are declared to be best) can be returned.

It appears that PSICA trees providing the best accuracies are obtained when the amount of splitting variables in the corresponding random forest is equal to the total amount of inputs. There is also an indication of that bootstrapping random forests instead of using a bias‐corrected infinitesimal jackknife might lead to lower uncertainties of the PSICA method. However, the price for this is greatly increased computational time. Some of the results also indicate that PSICA trees might not be very sensitive to the error's distribution.

The PSICA trees are computed by estimating probabilities and loss functions in a statistically motivated manner, which leads to high accuracies and low suspect rates in our simulation experiments. A real case study justifies the validity of our method because the information provided by the PSICA tree is also confirmed by previous medical studies.

The PSICA trees presented in this paper have some limitations. Firstly, the PSICA method was described for real‐valued outcome variables only. We also assumed that *Y*(*x*,*τ*)=*f*(*x*,*τ*)+*ϵ*, where *ϵ* is independent between different treatment options of the same patient, but this independence assumption might not hold in practice. However, since for a patient with some characteristics *X*_*i*_, we only observe *Y*(*X*_*i*_,*t*_*i*_) and never observe any other *Y*(*X*_*i*_,*τ*) such that *τ*≠*t*_*i*_, the observed data distribution will not depend on possible correlations cor(*Y*(*X*_*i*_,*t*_*i*_),*Y*(*X*_*i*_,*τ*)). Since the observed data do not contain information on the magnitude of these correlations, we model *ϵ* as a term, which is independent between different treatment options of the same patient.

It was also assumed that randomized clinical trials data are used. Accordingly, a further research direction is to generalize the PSICA algorithm to categorical outcome scenarios and to investigate how it needs to be modified for nonrandomized trials. Additionally, investigating the possibility of postpruning instead of prepruning might lead to a decrease in the suspect rates of the PSICA method.

## 1

**Table A1:** Mean accuracy rates (over 200 experiments) for different data models (M1 to M6) processed by four methods (m_1_ to m_4_). Standard error of the mean is specified in parentheses

n	model	*m*_1_	*m*_2_	*m*_3_	*m*_4_
300	1	1.00 (<0.001)	1.00 (<0.001)	1.00 (<0.001)	0.96 (0.006)
900	1	1.00 (<0.001)	1.00 (<0.001)	–	1.00 (0.001)
1800	1	1.00 (<0.001)	1.00 (<0.001)	–	1.00 (<0.001)
300	2	0.99 (0.001)	1.00 (<0.001)	0.97 (0.002)	0.78 (0.013)
900	2	1.00 (<0.001)	1.00 (<0.001)	–	0.94 (0.005)
1800	2	1.00 (<0.001)	1.00 (<0.001)	–	0.95 (0.003)
300	3	0.99 (0.001)	1.00 (<0.001)	0.97 (0.003)	0.73 (0.016)
900	3	1.00 (<0.001)	1.00 (<0.001)	–	0.93 (0.006)
1800	3	1.00 (<0.001)	1.00 (<0.001)	–	0.95 (0.004)
300	4	1.00 (<0.001)	1.00 (<0.001)	1.00 (0.001)	0.60 (0.009)
900	4	1.00 (0.001)	1.00 (<0.001)	–	0.82 (0.014)
1800	4	0.99 (<0.001)	1.00 (<0.001)	–	0.98 (0.005)
300	5	0.98 (0.002)	0.99 (0.001)	0.97 (0.002)	–
900	5	0.98 (0.002)	1.00 (<0.001)	–	–
1800	5	0.97 (0.002)	1.00 (<0.001)	–	–
300	6	0.93 (0.007)	0.88 (0.016)	0.62 (0.019)	–
900	6	0.90 (0.003)	0.90 (0.013)	–	–
1800	6	0.88 (0.003)	0.91 (0.013)	–	–

**Table A2:** Mean uncertainty rates (over 200 experiments) for different data models (M1 to M6) processed by four methods (m_1_to m_4_). Standard error of the mean is specified in parentheses

n	Model	*m*_1_	*m*_2_	*m*_3_	*m*_4_
300	1	0.36 (0.008)	0.48 (0.014)	0.14 (0.012)	0.87 (0.020)
900	1	0.29 (0.009)	0.58 (0.014)	–	0.99 (0.003)
1800	1	0.26 (0.008)	0.71 (0.014)	–	1.00 (<0.001)
300	2	0.23 (0.009)	0.49 (0.003)	0.17 (0.010)	0.42 (0.014)
900	2	0.20 (0.007)	0.50 (0.001)	–	0.51 (0.008)
1800	2	0.25 (0.006)	0.50 (0.001)	–	0.50 (0.007)
300	3	0.29 (0.010)	0.50 (0.003)	0.20 (0.011)	0.44 (0.019)
900	3	0.24 (0.008)	0.50 (0.001)	–	0.51 (0.008)
1800	3	0.27 (0.007)	0.50 (0.001)	–	0.50 (0.007)
300	4	0.41 (0.004)	0.43 (0.002)	0.43 (0.002)	0.34 (0.011)
900	4	0.26 (0.009)	0.44 (0.001)	–	0.50 (0.017)
1800	4	0.14 (0.007)	0.44 (0.001)	–	0.66 (0.007)
300	5	0.45 (0.009)	0.67 (0.008)	0.52 (0.009)	–
900	5	0.39 (0.009)	0.77 (0.010)	–	–
1800	5	0.35 (0.008)	0.88 (0.010)	–	–
300	6	0.40 (0.004)	0.42 (0.002)	0.38 (0.007)	–
900	6	0.22 (0.008)	0.42 (0.001)	–	–
1800	6	0.09 (0.004)	0.42 (0.001)	–	–

**Table A3:** Mean suspect rates (over 200 experiments) for different data models (M1 to M6) processed by four methods (m_1_  to m_4_). Standard error of the mean is specified in parentheses

n	Model	*m*_1_	*m*_2_	*m*_3_	*m*_4_
300	1	0.06 (0.007)	0.04 (0.007)	0.13 (0.015)	0.32 (0.018)
900	1	0.06 (0.008)	0.04 (0.008)	–	0.17 (0.005)
1800	1	0.03 (0.008)	0.03 (0.005)	–	–
300	2	0.00 (<0.001)	0.00 (<0.001)	0.00 (0.001)	0.12 (0.014)
900	2	0.00 (<0.001)	0.00 (0.001)	–	0.02 (0.003)
1800	2	0.00 (<0.001)	0.00 (0.001)	–	0.01 (0.002)
300	3	0.00 (<0.001)	0.00 (0.001)	0.01 (0.002)	0.11 (0.013)
900	3	0.00 (<0.001)	0.00 (0.001)	–	0.02 (0.003)
1800	3	0.00 (<0.001)	0.00 (0.001)	–	0.02 (0.003)
300	4	0.01 (0.004)	0.00 (<0.001)	0.06 (0.010)	0.49 (0.012)
900	4	0.00 (0.001)	0.00 (<0.001)	–	0.23 (0.011)
1800	4	0.00 (<0.001)	0.00 (<0.001)	–	0.10 (0.006)
300	5	0.00 (<0.001)	0.01 (0.003)	0.04 (0.005)	–
900	5	0.00 (<0.001)	0.01 (0.003)	–	–
1800	5	0.00 (0.001)	0.01 (0.003)	–	–
300	6	0.08 (0.013)	0.05 (0.015)	0.11 (0.018)	–
900	6	0.01 (0.003)	0.00 (0.001)	–	–
1800	6	0.00 (<0.001)	0.00 (<0.001)	–	–

**Table A4:** Mean decision accuracy rates (over 200 experiments) for different data models (M1 to M6) processed by PSICA methods (m_1_  to m_3_). Standard error of the mean is specified in parentheses

n	Model	*m*_1_	*m*_2_	*m*_3_
300	1	0.89 (0.003)	0.89 (0.003)	0.97 (0.003)
900	1	0.92 (0.003)	0.87 (0.003)	–
1800	1	0.94 (0.002)	0.84 (0.003)	–
300	2	0.96 (0.001)	0.89 (0.002)	0.97 (0.002)
900	2	0.97 (0.001)	0.88 (0.002)	–
1800	2	0.97 (0.001)	0.88 (0.002)	–
300	3	0.95 (0.002)	0.89 (0.003)	0.96 (0.002)
900	3	0.96 (0.001)	0.88 (0.002)	–
1800	3	0.96 (0.001)	0.87 (0.002)	–
300	4	0.89 (0.003)	0.80 (0.002)	0.85 (0.002)
900	4	0.95 (0.001)	0.83 (0.001)	–
1800	4	0.97 (0.001)	0.84 (0.001)	–
300	5	0.87 (0.003)	0.75 (0.003)	0.80 (0.004)
900	5	0.90 (0.002)	0.74 (0.002)	–
1800	5	0.90 (0.002)	0.72 (0.002)	–
300	6	0.85 (0.003)	0.78 (0.002)	0.80 (0.002)
900	6	0.95 (0.001)	0.82 (0.001)	–
1800	6	0.95 (0.001)	0.83 (0.001)	–

**Table A5:** Mean efficiency metrics (over 400 experiments) for different data models and data sizes processed by m_1_ with different α values. Standard error of the mean is specified in parentheses

***α***	Accuracy	Decision accuracy	Suspect	Uncertainty
10^−5^	0.982 (0.002)	0.932 (0.002)	0.000 (<0.001)	0.316 (0.008)
0.01	0.980 (0.002)	0.936 (0.002)	0.004 (0.001)	0.291 (0.007)
0.05	0.979 (0.002)	0.937 (0.002)	0.010 (0.003)	0.272 (0.007)
0.5	0.977 (0.002)	0.938 (0.002)	0.024 (0.004)	0.266 (0.007)
1	0.977 (0.003)	0.937 (0.002)	0.020 (0.003)	0.264 (0.007)
